# Protection of Concrete Structures: Performance Analysis of Different Commercial Products and Systems

**DOI:** 10.3390/ma14133719

**Published:** 2021-07-02

**Authors:** Denny Coffetti, Elena Crotti, Gabriele Gazzaniga, Roberto Gottardo, Tommaso Pastore, Luigi Coppola

**Affiliations:** 1 Department of Engineering and Applied Sciences, University of Bergamo, 24044 Dalmine (BG), Italy; denny.coffetti@unibg.it (D.C.); elena.crotti@unibg.it (E.C.); gabriele.gazzaniga@guest.unibg.it (G.G.); tommaso.pastore@unibg.it (T.P.); 2 UdR “Materials and Corrosion”, Consorzio INSTM, 50121 Florence, Italy; 3 Consorzio Interuniversitario per lo Sviluppo dei Sistemi a Grande Interfase, CSGI, 50019 Sesto Fiorentino, Italy; 4 Master Builders Solution SPA Italia, 31100 Treviso, Italy; roberto.gottardo@mbcc-group.com

**Keywords:** coatings, concrete, durability

## Abstract

The increasing demand for reconstructions of concrete structures and the wide availability on the market of surface protective products and systems could lead to misunderstandings in the decision of the most effective solution. Surface protectors have become increasingly widespread in recent years in concrete restoration interventions thanks to their properties: they are able to protect the substrate from aggressive agents and consequently extend the useful life of the structures. The aim of this article is first of all to present the surface protective treatments available on the market, outlining their strengths and weaknesses. Subsequently, a characterization of seven different commercial coatings for reinforced-concrete structures is provided, taking into account chemical nature, fields of use and effectiveness, both in terms of physic and elastic performance and resistance to aggressive agents that undermine the durability of the treated concrete elements.

## 1. Introduction

In recent years, the concept of sustainability has gained ground in the construction sector, and a particular attention has been paid to the durability of the structures [1,2]. In fact, with the same environmental impact deriving from the production, the building materials capable of ensuring a prolonged service life, even if characterized by a strong environmental impact deriving from their production, could be much more sustainable than “green” materials, whose durability is often unknown [3,4]. For this reason, one of the main strategies toward sustainability of building materials is to increase their durability, thus reducing the economic and environmental cost associated with repairing works or, worse, demolition and reconstruction. 

The penetration of aggressive external agents such as carbon dioxide or chlorides is what is mainly responsible for the deterioration of reinforced-concrete structures [5,6,7]. For new buildings, the durability of concrete elements can be guaranteed with proper mix design and construction details; in the case of existing structures with durability deficiency, these problems must be solved in a different way, such as cathodic protection/prevention, electrochemical-based techniques, migrant corrosion inhibitors and surface treatments. Cathodic protection/prevention is generally applied only to infrastructures; it requires periodic inspection and proper design to avoid insufficient protection or overprotection and related adverse side effects such as hydrogen embrittlement and stress corrosion cracking [8,9]. So far, electrochemical methods used for concrete re-alkalinization or chloride removal have shown controversial results and are relatively expensive [10,11]. The effect of migrant corrosion inhibitors in prolonging the service life of reinforced-concrete structures is not fully understood even if some recent findings seem to be very promising [12,13]. On the contrary, concrete surface treatments are widely used to delay the degradation process of reinforced-concrete structures, and their effectiveness in preventing the ingress of aggressive external agents has been extensively confirmed since the 1980s [14,15,16].

The aim of this paper is to outline the main protective surface treatments by analyzing their characteristics and to define, through a careful analysis of the properties, the selection criteria for each protective product or system in relation to the field of use and the expected performance. 

### 1.1. Available Surface Treatments

To date, the surface treatments can be divided in three different macrocategories, in accordance with European Standard EN 1504-2 [17]:

(a) Polymeric and cementitious coatings
(b) Hydrophobic impregnations
(c) Pore-blocking treatments

#### 1.1.1. Polymeric and Cementitious Coatings

The surface coatings are defined as continuous films able to act as a physical obstacle to limit the penetration of aggressive substances into concrete elements. There are basically two different types of surface coatings: the polymer-based and cementitious protective coatings. The first ones promote the formation of a dense polymeric film with a thickness of about 0.1–1 mm on the concrete surface while the second ones realize a low-permeability cement-based layer with a thickness of about 2–10 mm. 

Traditional polymeric coatings have always been widely used in the construction sector. The main categories of polymeric coatings are epoxy resins [18,19,20], acrylics [21,22,23] and polyurethanes [24,25,26], each of them with their own strengths and weaknesses as reported in Table 1. They exert their protective ability by increasing the resistance against aqueous solutions penetration due to a barrier effect. In particular, the low water penetration of the coating can be obtained by modifying the water-polymer affinity and/or by modifying the microstructure, morphology and crosslink density of polymer with the aim of reducing the porosity of the coating. The main causes of failure of this coating type lie in the partial loss of adhesion with the substrate due to osmotic pressure (i.e., blistering), in the formation of microcracks due to the thermal shrinkage of the coating or crackling of the concrete and in peeling phenomena due to the penetration of aggressive substances into defects. Furthermore, several polymer coatings are characterized by a poor ultraviolet light (UV) resistance, and thus, their durability in outdoor environments could be limited. Recently, several studies are showing that the use of nanoparticles in polymers can enhance the mechanical strength, the physical properties and the protective capability of coatings even if the high costs and few applications in real structures limit the availability of nanocomposite-based polymer coatings on the market.

Cementitious coatings are cement-based mortars manufactured with a huge number of polymers (such as epoxy, polyurethane or acrylate) able to improve the mechanical properties (such as tensile strength, adhesion and resilience), the chemical resistance and the impermeability of cement mortars. The protective effects of cementitious coatings on concrete structures can be summarized as follow:

(a) The use of polymers strongly reduces the microcracks formation in the coatings, reducing the penetration of aggressive substances;
(b) The pore structure of polymer-containing cementitious mortars is finer than that of traditional cement-based composites, further improving the impermeability of coatings;
(c) The low-elastic modulus of the cementitious coatings enhances their crack-bridging capability, ensuring high performances even in the presence of cracked concrete.

Emerging solutions for concrete protection based on alkali-activated materials and geopolymers show rapid setting times, excellent bond strength and durability, low water and chloride permeability and high freeze-thaw resistances. Moreover, these new coatings also possess an electrolytic conductivity, which can allow them to act as a protective layer for concrete and skin sensor for structural health monitoring. To date, this kind of coating is still in the experimental phase (especially for issues related to the shrinkage reduction), but it is extremely promising and close to being widely used on real reinforced-concrete structures. 

#### 1.1.2. Hydrophobic Impregnation

Hydrophobic impregnations perform their task of avoiding the penetration of aggressive substances in liquid phase into the concrete by increasing the contact angle (the surface is considered hydrophobic when the contact angle is greater than 90° [27]) without modifying the water vapor transport within the cementitious matrix. The most common hydrophobic impregnation is based on silanes or siloxanes, small molecules (in the range of 1–7 nm) able to easily penetrate in the concrete pores and reduce the surface tension of the concrete. The characteristics of the alkyl group define the properties of the impregnating agent: the greater the molecular weight of the alkali group, the greater the degree of hydrophobicity of the treatment. By contrast, the characteristics of the alkoxy group are directly associated with the penetration depth of the hydrophobics. These surface treatments are very effective in limiting the ingress of aggressive aqueous solutions in the concrete, but at the same time, the dry pores could favor the carbonation of the cement matrix. In this way, in case of a deficiency in the surface treatment and wet environment, the carbonated concrete could be prone to steel corrosion.

#### 1.1.3. Pore Blocking Treatments

Impregnating coatings (i.e., pore-blocking treatments) are able to block the capillary pores of the concrete and consequently improve the impermeability of buildings [28,29]. Generally, the treatments available on the market are based on silicates (such as lithium silicate, sodium silicate and calcium silicate) that, in contact with the chemical constituents of concrete, form a colloidal gel capable of blocking the pores and avoiding the penetration of potentially harmful external agents. In addition to the abovementioned techniques, several studies are in progress on innovative precipitations techniques involving the use of bacteria [30,31] or dimethyl carbonate solutions [32]; however, to date, they are expensive and difficult to control/apply, and some issues are not fully understood. 

### 1.2. Main Properties of Surface Treatments

The choice of the ideal surface treatment strongly depends on the purpose of the work: protection against the ingress of aggressive substances into concrete and protection against the physicomechanical actions that occur during the service life of a structure or both. The following are the main properties of the surface treatments commercially available for concrete protection.

#### 1.2.1. Physical Parameters

First of all, it must be stated that the mechanical performance of the substrate cannot be improved in relation to the performance of the treatment, since the latter is not able to modify the porosity of the concrete; however, it is able to prevent the deterioration of concrete performance. In this regard, the compressive strength is not a noteworthy parameter in the examination of the treatments. 

A more important parameter for the surface treatments is the bonding strength; good adhesion to the substrate ensures its long-term performance and protection of concrete [33]. Experimental investigation [34] showed that the average bond strength of different epoxy coatings ranges between 2.9 and 4.0 MPa, and for cementitious mortar, it is about 0.8–1.2 MPa. The adhesion could be influenced by different aspects, such as the utilization of a primer, the roughness and the quality of the substrate and the application technique [35]. It is known that the adhesion property is strongly conditioned by aging of the treatment; unfortunately, no significant number of studies have been conducted in order to understand how much age influences the adhesion property and what the problems related to the aging of the same are [36,37].

Another important indicator of the effectiveness of a treatment is the abrasion resistance, since it is able to evaluate of the service life of surface-treated concrete under repetitive traffic loadings [38,39]. Many of surface treatments can improve the wear resistance of concrete surface. It is important to understand the mechanism according to which the concrete is protected from abrasion since it varies by the treatment type. Dang et al. [40] found that most organic surface coatings could improve the abrasion resistance of concrete, especially epoxy, while methacrylate with a high molecular weight showed no protection. A slight enhancement of abrasion resistance was observed for the concrete treated with silanes. Franzoni et al. [41] investigated the effects of some inorganic surface treatments on abrasion resistance and found out that sodium silicate showed the best effect because it could form a protective layer with a remarkable thickness. 

The crack-bridging ability is the property of the coatings to cover cracks formed in the concrete substrate [42], keeping the properties intact and reducing the risk that cracks can propagate and cause deterioration. The crack-bridging ability is closely related to the type of protective material chosen and its elasticity characteristics: the polymer-cement coatings have an excellent crack bridging ability, making this type of treatment suitable for application on cracked supports; the reduced elasticity of epoxy resins and acrylic coatings makes ineffective the crack-bridging properties, reducing the range of applications of the latter to sound substrates only [43,44,45].

The resistance of the treatment against possible alterations promoted by the environment in which they are located or by the tasks they must perform is very important for the success of a protection intervention. Research showed that temperature and ultraviolet radiation highly affected the efficiency of surface treatments. Vries [46] studied resistance of hydrophobic surface treatments, such as silane and siloxane, at the high temperature. The results showed that the water absorption rate for the treated concrete increased dramatically after half-an-hour storage in a 160 °C chamber. Levi et al. [47] found that protection of silane, silicone and fluorinated polymer on concrete water absorption decreased by 50% after ultraviolet aging; by contrast, the polymer-modified cementitious coatings have a great ultraviolet light resistance, which allows for their use in direct contact with sunlight. 

#### 1.2.2. Durability Parameters

In most cases, the success of the protection is related to the durability of the concrete-surface treatment systems [48,49]. Since many aggressive substances are transported through water or air, the permeation characteristics of the surface concrete is an important factor for the durability of whole concrete element [50]. Many surface coatings are able to reduce the ingress of water through the treated matrix. The best treatments from the point of view of water permeability are epoxy coatings, silane with an acrylic top coat, methyl methacrylate, and alkyl alkoxysilane [51]. Almusallam et al. [52] found that the uncoated cement mortars absorbed water at a very rapid rate, and after 56 h, the total absorption was about 5% by weight; after being treated with polymer emulsion, acrylic, chlorinated rubber, polyurethane coatings, and epoxy coatings, the absorption of water decreased to 3.3–3.4%, 0.23–1.46%, 0.76–1.04%, 0.21–1.83%, and 0.27–1.3%, respectively. Moreover, Medeiros et al. [53,54] demonstrated that silane and siloxane have a good capability of inhibiting water penetration as long as the water pressure was lower than 12,000 Pa. The results indicated that hydrophobic surface treatments should only be used when the water exposure conditions are well known. Modified cementitious mortar coating, on the other hand, have slightly higher resistance to water penetration than the other treatments; despite this, the modified cementitious mortar coatings are highly recommended in the protection of structures in everlasting contact with water because, unlike other treatments, they have a greater resistance to leaching [55,56].

In a concrete structure, chlorides can penetrate into the cement matrix and initiate degradation phenomena due to diffusion under the influence of a concentration gradient, migration in an electrical field or absorption due to a capillary action [57]. In most cases, the protective treatments are able to reduce the concentration of chlorides in the substrate. Unfortunately, both for the great variety of treatments and test methods, it is difficult to identify the best treatment even if polymer coatings seem to exhibit the higher protection against chloride ingress in the cementitious matrix. Almusallam et al. affirmed that the polyurethane and acrylic coatings are able to increase the resistance against the diffusion of chloride ions of about 10 times compared to the uncoated concrete [52]. Brenna et al. [58] found the good behavior of polymer modifies cementitious coatings also after 17 years of exposure, highlighting also the influence of the polymer content on the properties of coatings. According to Coppola et al. [12], the silane-based treatment is able to significantly reduce the penetration of chlorides into the matrix, regardless of the type of cement used.

Especially in urban or industrialized areas, the problem of carbon dioxide penetration is relevant. Carbonation is a chemical reaction between Ca(OH)_2_, calcium-silicate-hydrate (C-S-H) and CO_2_ to form CaCO_3_, silica-rich C-S-H and amorphous silica gel [59]; it is able to destroy the passivity of the embedded reinforcement bars and to promote corrosive phenomena [60,61]. The factors controlling carbonation are the diffusivity of CO_2_ and the reactivity of CO_2_ with the concrete. They depend on the pore system of hardened concrete and the exposure condition [62]. Many studies agree that acrylic coatings are the best choice to prevent the penetration of carbon dioxide, while treatments based on silanes or siloxanes can control the humidity of the concrete substrate but are not able to reduce the permeability to air and consequently not even the penetration of carbon dioxide [63,64,65].

Although the treatments cannot fulfil the functions performed by the air-entraining additives against the phenomena related to freezing and thawing cycles, they can contribute to making the concrete substrate more resistant to cold climate by preventing the critical moisture content from being reached. Both the acrylic treatments and the polymer-modified cementitious coatings have a good behavior against the destructive phenomena induced by the freezing and thawing cycles; by contrast, the behavior of silane-based coatings has not yet been well defined and is debated by the scientific community: Basheer et al. [66] stated that the silane treatment could double the number of freeze–thaw cycles at which concrete began cracking in fresh water test; other studies show that silane-treated concrete deteriorates more quickly than untreated concrete in laboratory accelerated freeze-thaw tests. This type of treatment would be very effective if the concrete substrate would be completely dry since they would not allow water to penetrate; however, in reality the substrate is never completely dry and therefore the silane-based treatment is not able to reduce the risk associated with freezing and thawing curls [67,68].

## 2. Experimental Part

### Materials and Methods

In order to provide a complete analysis able of evaluating some of the aspects previously described, 7 different commercial treatments (Table 2) have been selected and tested in order to outline a guideline for selecting the best surface treatment in accordance with the expected purposes. The products investigated are: a water-based acrylic protective (A), a water-based acrylic elastomeric protective (AE), an epoxy coating (E), an epoxy-bituminous coating (EB), an epoxy-polyurethane coating (EP), a polyurethane coating (P) and a polymer-modified cementitious coating (PMC). The treatments were applied on a concrete substrate manufactured in accordance with EN 1766 [69] (CEM I 42.5 R, water-to-cement ratio 0.40, natural aggregates with maximum size 10 mm and superplasticizer in compliance with EN 934-2 [70]). The summary of the experimental tests carried out is reported in Table 3. 

## 3. Results

### 3.1. Pot Life

The pot life is defined as the maximum time during which a coating material supplied as separate components (product A and product B or powder and water) should be used after the components have been mixed together. This parameter was evaluated at room temperature by taking into account the longest period of time which the consistency met the requirements related to the laying of the coating. Results reported in Table 4 evidenced that the average pot life at room temperature of the surface treatments is close to 1 h with the exception of P, which is applicable for about 3 h after mixing. However, it is necessary to point out that the pot life is strongly affected by the job-site temperature: the higher the temperature, the shorter the pot life. 

### 3.2. Adhesion

The adhesion tests were carried out on samples before and after thermal cycles in presence of deicing salts. In particular, after the curing of concrete and the application of the protective coating, a core drilling is carried out that affects both the substrate and the protective one. Then, a plug is applied, and finally, at a speed of 0.05 MPa/s, the tear test is performed on one half of samples to evaluate the adhesion and the failure type. On the other hand, the other specimens were subjected to 50 thermal cycles (immersion in a saturated sodium chloride solution at a temperature of −15 ± 2 °C for 2 h, followed by immersion in water at a temperature of 21 ± 2 °C for 2 h) before the adhesion test. 

**Figure 1:**
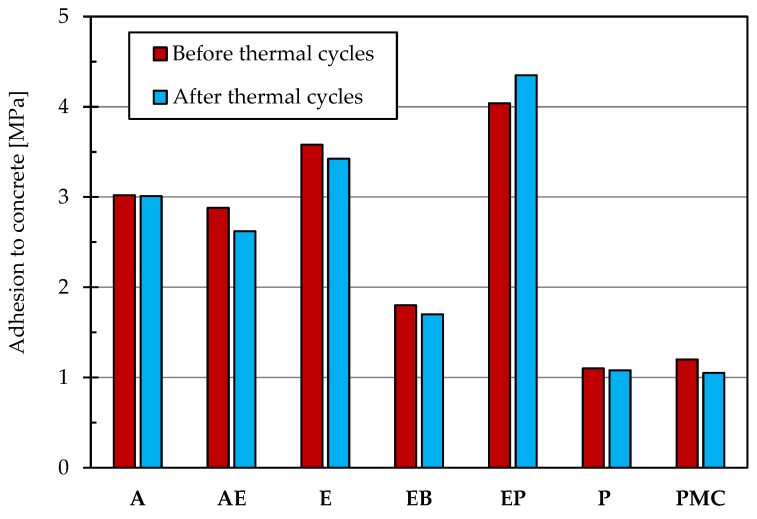
Adhesion to concrete before and after 50 freeze/thaw cycles with deicing salts.

Figure 1 shows the adhesion strength of the tested treatments in both the aforementioned cases. According to Garbacz [33], the results belong to the studied range; moreover, it is possible to observe that the EP and E coatings have the best adhesion both before and after the thermal cycles with failure on concrete substrate, acrylic and acrylic elastomeric products are characterized by adhesion close to 3 MPa with failure on the coating/concrete interface and EB, P and PMC show similar adhesion values with different failure mechanism: the failure of epoxy-bitumen coating was on the coating/concrete interface, while the polyurethane and polymer-modified cementitious coatings exhibited a cohesive failure.

### 3.3. CO_2_ and Water Vapor Permeability

The resistance to carbon dioxide diffusion is an important parameter in the choice of a coating. To avoid any design errors or the impossibility of carrying out the restoration work in a workman-like manner, it is possible to integrate the missing concrete cover thickness by choosing a suitable protective. In light of this, EN 1504-2 [17] sets a minimum value of resistance to carbon dioxide equal to 50 m of equivalent air thickness; most of the coatings on the market, to operate in favor of safety, have values that are much higher than those required by the standard for a coating. In addition to the diffusion of carbon dioxide, it is necessary to prescribe a minimum value for the resistance offered to vapor diffusion. This need arises from the fact that if on the back of the coating the cement matrix is saturated or partially saturated with water, when this reaches the concrete/coating interface, it could be transformed into water vapor due to solar radiation and thus determining the swelling or the detachment of the vapor-tight coat. To avoid these problems, EN 1504-2 [17] requires for vapor diffusion values to be less than 5 m of equivalent air thickness. 

**Figure 2:**
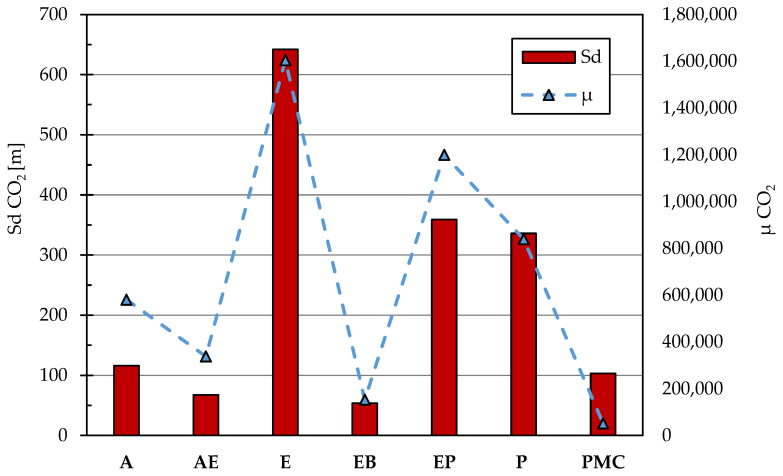
Resistance against CO_2_ permeability of coatings expressed in terms of Sd CO_2_ (left) and μ CO_2_ (right). Sd CO_2_ is calculated as the product of μ CO_2_ and the application thickness (t).

The results, first of all, highlight that all the protectives tested comply with the limits prescribed by EN 1504-2 [17] for the protection of reinforced-concrete structures. In particular, Figure 2 evidenced that E, EP and P are impervious to carbon dioxide (Sd CO_2_ greater than 300 m), epoxy-bitumen and acrylic elastomeric-based treatments have a resistance to CO_2_ penetration of just over 50 m. At the same time, in this study, the E and PMC are characterized by an Sd CO_2_ close to 100 m of equivalent air thickness. On the other hand, authors want to highlight that unpublished experimental results on polymer-modified cementitious coatings demonstrated that the Sd CO_2_ is strongly influenced by the type and dosage of polymer used in manufacturing the coating. In particular, it is possible to obtain carbon dioxide resistances up to those of polyurethane-based coatings. 

**Figure 3:**
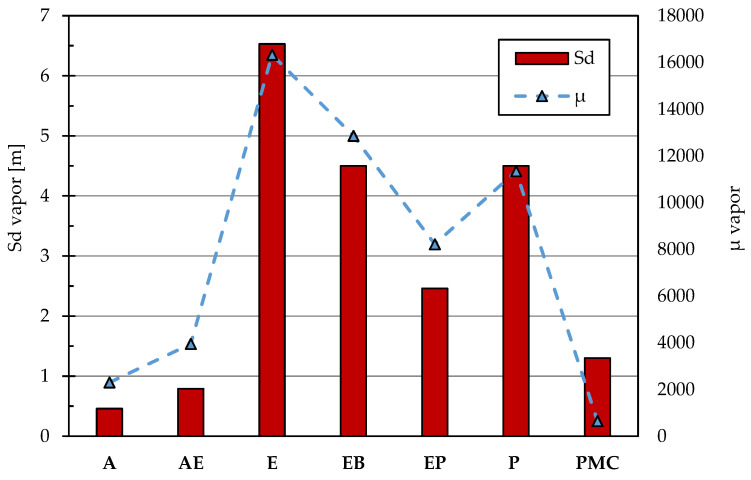
Resistance against water vapor permeability of coatings expressed in terms of Sd vap (left) and μ vap (right). Sd CO_2_ is calculated as the product of μ vap and the application thickness (t).

In terms of water vapor permeability (Figure 3), the best performance is offered by the polymer-modified cementitious coating that has the lowest values together with acrylic coatings. To the detriment of other excellent performances, epoxy coatings can suffer from low vapor permeability which strongly increase, in the presence of wet concretes and/or exposure to solar radiation, the risk of detachment and blistering of the coating [83,84].

### 3.4. Capillary Absorption

Aggressive substances for the cement matrix are often transported by water by capillary absorption. For this reason, the EN 1504-2 [17] standard limits the water capillary absorption of coated concrete by setting the maximum capillary absorption of coating to 0.1 kg/m^2^h^0.5^. Results reported in Table 5 show that all the products investigated meet the standard requirements. In particular, epoxy-based coats (E, EB and EP), similarly to polyurethane products, are characterized by a negligible water absorption (0.001–0.008 kg/m^2^h^0.5^), while A and AE treatments evidence a capillary absorption coefficient close to the standard limits of 0.1 kg/m^2^h^0.5^. By contrast, the waterproofing ability of polymer-modified cementitious coatings are lower with respect to the epoxy-based treatments, even if it is necessary to demonstrate that this property is also strongly affected by the type and dosage of polymer used in manufacturing the coating. In particular, a proper composition allows one to reach extremely low capillary absorption coefficients.

### 3.5. Crack-Bridging Ability

**Figure 4:**
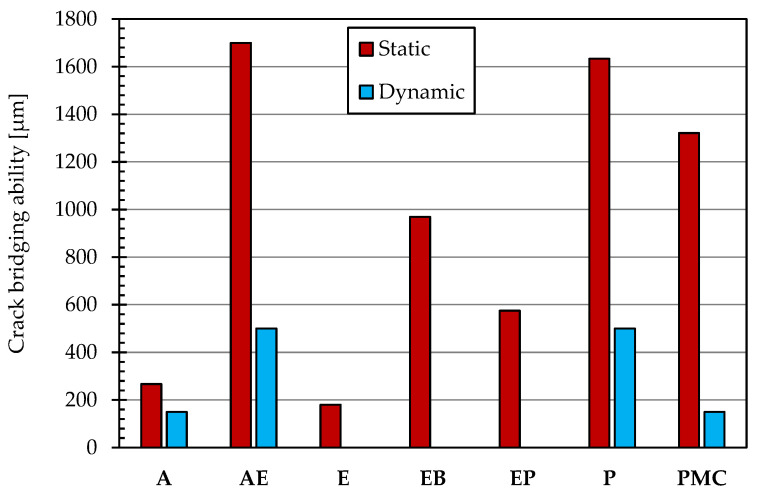
Crack-bridging test results.

The ability to cover the cracks that occur in the substrate to avoid the penetration of aggressive agents turns out to be a fundamental parameter in the choice of a protective. The graph reported in Figure 4 highlights the static crack-bridging values at a temperature of 23 °C in compliance with EN 1062-7 [77]. The AE and P coatings show an excellent ability to cover cracks wider than 1.6 mm; PMC crack-bridging ability is close to 1300 μm, while EB limits their static crack bridging at about 1 mm. By contrast, the intrinsic lack of elasticity of epoxy and acrylic systems is evident in this test. Finally, the epoxy-polyurethane coating is only able to bridge cracks with a width lower than 600 μm. 

In addition to the static crack-bridging ability, the capability of the coatings to take up the elongation resulting from the periodic movement of the crack sides was evaluated for different crack widths. After 1000 cycles with a maximum crack width of 500 μm, AE and P appear to be sound, while the A and PMC systems limit their dynamic ability to cover cracks of about 150 μm (Figure 4). On the other hand, the epoxy nature of the other systems (E, EP, EB) does not guarantee adequate dynamic crack-bridging ability. 

### 3.6. Abrasion Resistance

In relation to the weight loss connected to a forced abrasion (EN 5470-1 [78]) listed in Table 5, it is possible to note that epoxy, polyurethane and epoxy-polyurethane protective products are more resistant to abrasion (mass loss in the range of 200–300 g at the end of the test), while PMC and EB coatings show a weight loss that is about one order of magnitude higher than the aforementioned systems. The reason for this strong damage following an abrasive tickling is to be found in the physical properties of the coatings; in fact, as previously highlighted by the capillary absorption tests, PMC and EB are more porous, therefore less compact and consequently more exposed to these stresses [40,41]. 

### 3.7. Resistance to Severe Chemical Attack

The evaluation of the resistance to make contact with chemical agents is an important parameter closely related to the intended applications of the coating; due to these peculiarities, some protective products have not been tested because their low resistance against chemicals is commonly known [85]. Table 6 shows the hardness values (EN ISO 868 [86]) after the exposure to different aggressive chemical agents in accordance with UNI 13529 [79]. Results are expressed from 0 to 100: a high shore hardness means a great resistance to chemicals exposure. The treatment that shows greater protection against aggressive chemicals is the EP one, since, following exposure to chemicals, it reports hardness values that are on average 5% higher than the other coatings analyzed. E and MPC coatings also guarantee good chemical resistance [87,88], even if it should be noted that cement polymer-modified coatings show significant deterioration when exposed to acidic environments [89]. In fact, following an exposure to 10% aqueous acetic acid and 20% sulfuric acid, the total dissolution of the coating layer takes place.

### 3.8. Resistance to Hydraulic Pressure

The analysis of the parameter of resistance to negative hydrostatic pressure becomes of primary importance for waterproofing underground structures such as retaining walls. For this purpose, not all the tested coatings are suitable; therefore, the test in compliance with EN 8298-8 [80] was conducted only on E, EP and MPC coatings. These coatings, following the application of different hydrostatic pressures (2, 5, 10, 50, 100, and 250 kPa for 72 h), did not show any state of alteration connected to the permeation of pressurized water, making them suitable for this purpose. 

### 3.9. Fire Resistance

The fire resistance is a fundamental properties of building materials, and it was evaluated by determining the ignitability of products by direct flame impingement and the production of smoke and flaming droplets. Experimental results reported in Table 7 evidenced a limited resistance to fire of PMC and EB, because they contain huge number of inflammable polymers (PMC) or oil-derived products (EB). On the other hand, acrylic elastomeric, epoxy, epoxy-polyurethane and polyurethane coatings showed a moderate fire resistance while acrylic product is characterized by a high resistance to flames. Moreover, all the specimens (except EB and PMC) are characterized by a low smoke production (class s1) and no formation of flaming droplets and particles (class d0). However, it is necessary to outline that the fire resistance of polymer-modified cementitious coatings is strongly influenced by the type and the dosage of the polymer used; in general, the fire resistance class of PMC is in the range F–C (class A1 indicated the maximum fire resistance, class F the minimum). 

### 3.10. Resistance to UV Light and Moisture

All the samples were exposed to wetting and drying cycles and UV radiation in accordance with EN 1062-11 [82] to determine the resistance of coatings to UV light and moisture. After more than 1000 h of exposure (about 125 wetting and drying cycles), no cracks, detachments and flaking were found. 

## 4. Conclusions

In light of the scientific literature review and tests performed on protective products, the following conclusions can be drawn:

(a) Water-based acrylic and water-based acrylic elastomeric coatings are the best systems when it is necessary to maintain the original texture and/or a specific aesthetic finish is desired. Acrylics have an excellent resistance to CO_2_ penetration and a proper water absorption; they usually do not have excellent crack-bridging capabilities and, due to their conformation, are not suitable for withstanding mechanical stress or contact with aggressive chemical agents. They can be used to protect concrete in structures not fully immersed in water or in contact with aggressive chemicals; finally, elastomeric acrylic coatings embrace a slightly wider market share given the crack-bridging properties that make them suitable for applications where an elasticity of the coating is required, such as the protection of residential buildings.
(b) Polyurethane and epoxy resin-based coatings, whether they are epoxy, epoxy-polyurethane or epoxy-bituminous can be used to protect concrete structures subject to continuous contact with water and aggressive chemicals. The main applications of this family of coatings are the protection a of reinforced-concrete structures subjected to severe environmental aggressions such as sewage collectors, purification plants (settling and aeration tanks), and bridge decks. The limits of epoxy protectives are the high resistance to vapor diffusion which could compromise the durability of coatings applied on wet substrates and the reduced crack-bridging capability that preclude their use on cracked concretes. The EB, EP and P, on the contrary, shows good crack-bridging ability, which makes them suitable for applications where a good ability to cover cracks is required, such as waterproofing of canals or containment tanks.
(c) Polymer-modified cementitious coatings have excellent resistance to steam penetration and crack-bridging ability, as well as a high resistance to hydraulic pressure. They can be applied in the protection of both new concrete structures or during restoration works. The high waterproofing properties make these products suitable for the protection of reinforced-concrete structures such as tanks, fountains, wells, flower boxes or in any case of structures in continuous contact with water without extremely aggressive chemical agents. Their elasticity makes these protective products suitable for under-tile applications typical of the restoration works of projecting structures such as terraces and balconies. Therefore, it is necessary to highlight that the main properties of these coatings are strongly related to the type and dosage of polymer used during their manufacturing.

## Figures and Tables

**Table 1:** Advantages and disadvantages of different surface treatments (classification according to the authors’ experience and by analyzing the principal products available on the European market and common best practices).

Properties	Acrylic	Polyurethane	Epoxy Resins	Cementitious Coatings
Thickness	50–300 µm	200–400 µm	>400 µm	2–3 mm
“Barriers” ability	Medium	High	Very high	Variable *
Aesthetic conservation	Excellent	Very good	Sufficient	Low
Vapor permeability	High	Medium	Low	High
Crack-bridging ability	Excellent	Good	Low	Variable *
UV resistance	Excellent	Excellent	Medium	Excellent
Freeze/thaw resistance	Excellent	Excellent	Excellent	Good
Durability in continuous contact with water	Low	Low	Excellent	Excellent
Application at low temperature	Good	Good	Low	Good
Application on wet concretes	Medium	Low	Low	Excellent

* These properties vary as a function of the type and dosage of polymer used to produce the cementitious coating.

**Table 2:** Properties of surface treatments.

Treatment	Density	Solids	Thickness (t)
Water-based acrylic (A)	1.54 kg/dm^3^	74%	200 µm
Water-based acrylic elastomeric (AE)	1.35 kg/dm^3^	63%	200 µm
Epoxy coating (E)	1.50 kg/dm^3^	84%	400 µm
Epoxy-bituminous (EB)	1.00 kg/dm^3^	42%	350 µm
Epoxy-polyurethane (EP)	1.30 kg/dm^3^	80%	300 µm
Polyurethane (P)	1.30 kg/dm^3^	58%	400 µm
Polymer-modified cementitious (PMC)	2.05 kg/dm^3^	75%	2 mm

**Table 3:** Details on the experimental tests carried out on coatings.

Test	Standard	A	AE	E	EB	EP	P	PMC
Pot life	EN ISO 9514 [71]	x	x	x	x	x	x	x
Adhesion to concrete	EN 1542 [72]	x	x	x	x	x	x	x
Adhesion to concrete after freeze/thaw cycles with deicing salts	EN 13687-1 [73]	x	x	x	x	x	x	x
CO_2_ permeability	EN 1062-6 [74]	x	x	x	x	x	x	x
Water vapor permeability	EN ISO 7783 [75]	x	x	x	x	x	x	x
Capillary water absorption	EN 1062-3 [76]	x	x	x	x	x	x	x
Crack-bridging ability (static and dynamic)	EN 1062-7 [77]	x	x	x	x	x	x	x
Abrasion resistance	EN ISO 5470-1 [78]			x	x	x	x	x
Resistance to severe chemical attack	EN 13529 [79]			x		x		x
Resistance to negative hydraulic pressure	UNI 8298-8 [80]			x		x		x
Fire resistance	EN 13501-1 [81] and related standards	x	x	x	x	x	x	x
UV light and moisture resistance	EN 1062-11 par. 4.2 [82]	x	x	x	x	x	x	x

**Table 4:** Pot life of coatings.

Properties	A	AE	E	EB	EP	P	PMC
Pot life [min]	60	60	45	90	60	180	60

**Table 5:** Capillary absorption coefficient and results of abrasion test.

Surface Treatment	Capillary Absorption Coefficient [kg/m^2^h^0.5^]	Mass Loss at the End of Abrasion Test [g]
A	0.090	–
AE	0.090	–
E	0.001	298
EB	0.008	2874
EP	0.006	238
P	0.003	254
PMC	0.010	2949

**Table 6:** Resistance to chemical aggression (hardness shore D).

Chemical	E			EP			PMC		
	0 Day	3 Days	28 Days	0 Day	3 Days	28 Days	0 Day	3 Days	28 Days
All hydrocarbons	73	73	77	78	85	84	76	80	75
All alcohols and glycol ethers	74	77	75	75	80	79	–	–	–
Hydrogenated hydrocarbons	74	76	76	74	N.D.	N.D.	–	–	–
Aqueous solutions of organic acids up to 10%	76	74	66	81	79	69	78	N.D.	N.D.
Inorganic acids up to 20% and acidic hydrolyzing salts in aqueous solutions	76	78	75	83	82	76	78	N.D.	N.D.
Inorganic bases and the alkaline hydrolyzing salts in aqueous solutions	73	78	77	80	79	82	78	76	77
Solutions of inorganic nonoxidizing salts	76	77	77	81	84	78	82	81	82
water at the inlet of the purifier	75	76	77	81	84	78	76	77	76

N.D. = Not detectable due to severe deterioration.

**Table 7:** Fire resistance of coatings (main results).

Surface Treatment	Classification	Direct Flame Test Results			
		Flame Spread	FIGRA *	SMOGRA **	Flaming Droplets
A ^#^	A2 s1 d0	≤150 mm within 60 s	≤120 W/s	≤30 m^2^/s^2^	No droplets
AE	B s1 d0	≤150 mm within 60 s	≤120 W/s	≤30 m^2^/s^2^	No droplets
E	C s1 d0	≤150 mm within 60 s	≤250 W/s	≤30 m^2^/s^2^	No droplets
EB	E	≤150 mm within 20 s	–	–	–
EP	B s1 d0	≤150 mm within 60 s	≤120 W/s	≤30 m^2^/s^2^	No droplets
P	B s1 d0	≤150 mm within 60 s	≤120 W/s	≤30 m^2^/s^2^	No droplets
PMC	E	≤150 mm within 20 s	–	–	–

* FIGRA (fire growth rate index) is the maximum of the quotient of heat release rate from the sample and the time of its occurrence using a total heat release threshold in the first 600 s of 0.4 MJ (for class C) or 0.2 MJ (for class B and A2); ** SMOGRA (smoke growth rate index) is the maximum of the quotient of smoke production rate from the sample and the time of its occurrence; # every class A2 product shall satisfy the same criteria as for class B with the addition of non-combustibility tests in accordance with EN ISO 1182 [90].

## Data Availability

Not applicable.
